# Successful high-level accumulation of fish oil omega-3 long-chain polyunsaturated fatty acids in a transgenic oilseed crop

**DOI:** 10.1111/tpj.12378

**Published:** 2013-11-08

**Authors:** Noemi Ruiz-Lopez, Richard P Haslam, Johnathan A Napier, Olga Sayanova

**Affiliations:** Department of Biological Chemistry and Crop Protection, Rothamsted ResearchHarpenden, AL5 2JQ, UK

**Keywords:** *Camelina sativa*, desaturase, elongase, omega-3, polyunsaturated fatty acids, transgenic plants

## Abstract

Omega-3 (also called *n*-3) long-chain polyunsaturated fatty acids (≥C20; LC-PUFAs) are of considerable interest, based on clear evidence of dietary health benefits and the concurrent decline of global sources (fish oils). Generating alternative transgenic plant sources of omega-3 LC-PUFAs, i.e. eicosapentaenoic acid (20:5 *n*-3, EPA) and docosahexaenoic acid (22:6 *n*-3, DHA) has previously proved problematic. Here we describe a set of heterologous genes capable of efficiently directing synthesis of these fatty acids in the seed oil of the crop *Camelina sativa*, while simultaneously avoiding accumulation of undesirable intermediate fatty acids. We describe two iterations: RRes_EPA in which seeds contain EPA levels of up to 31% (mean 24%), and RRes_DHA, in which seeds accumulate up to 12% EPA and 14% DHA (mean 11% EPA and 8% DHA). These omega-3 LC-PUFA levels are equivalent to those in fish oils, and represent a sustainable, terrestrial source of these fatty acids. We also describe the distribution of these non-native fatty acids within *C. sativa* seed lipids, and consider these data in the context of our current understanding of acyl exchange during seed oil synthesis.

## Introduction

**Figure 1:**
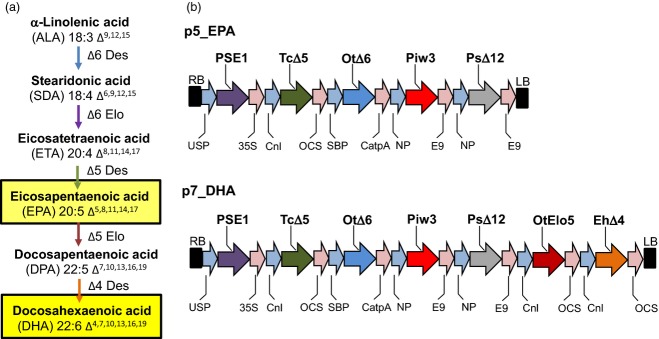
Production of EPA and DHA in *C. sativa*. (a) Biosynthetic pathway for the production of the LC-PUFAs EPA and DHA, with the various enzyme activities shown in different colours [also used in (b)]. (b) Simplified maps of vectors p5_EPA and p7_DHA used for transformation of *C. sativa*. Abbreviations: Cnl, conlinin 1 promoter for the gene encoding the flax 2S storage protein conlinin; USP, promoter region of the unknown seed protein of *Vicia faba*; SBP, sucrose binding protein 1800 promoter; NP, napin; OtΔ6, Δ6-desaturase from *O. tauri*; TcΔ5, a Δ5-desaturase from *Thraustochytrium* sp.; Piw3, ω3-desaturase from *Phytophthora infestans*; PsΔ12, a Δ12-desaturase from *Phytophthora sojae*; EhΔ4, Δ4-desaturase from *E. huxleyi*; PSE1, a Δ6-elongase from *P. patens*; OtElo5, Δ5-elongase from *O. tauri*; OCS, 35S, E9 and CatpA represent terminators.

It is now well established that omega-3 LC-PUFAs have critical roles in human health (Saravanan *et al*., 2010). Currently, the primary dietary source of these fatty acids is marine fish; however, the increasing demand for fish oil (particularly due to the expansion of the aquaculture industry, the major consumer of these oils) places enormous pressure on diminishing marine stocks (Cressey, 2009). Such over-fishing and concerns related to pollution in the marine environment have triggered an urgent search for a completely new source of omega-3 LC-PUFAs (Tocher, 2009). When evaluating any potential new source of these fatty acids, it should be both sustainable and capable of meeting any increased demand. An appealing approach to renewable supply of omega-3 LC-PUFAs is metabolic engineering of a crop plant with the capacity to synthesize these fatty acids in seeds. The scalability of agriculture-based production systems, in conjunction with modest running costs, highlights the potential of transgenic plants as ‘green factories’ for the synthesis of desired compounds (Dyer *et al*., 2008). An oilseed crop such as *Camelina sativa* is an attractive host for such metabolic engineering, based on its low input costs and ease of transformation (Nguyen *et al*., 2013). Introduction of the omega-3 LC-PUFA biosynthetic pathway into a crop demands the addition of multiple genes (for primary synthesis and to direct the flux of substrate and biosynthetic intermediates) that require co-ordinated tissue-specific expression in the developing seeds of the transgenic host (Cheng *et al*., 2010; Haslam *et al*., 2013). It is for this reason that reconstruction of this pathway represents the cutting edge of metabolic engineering in transgenic plants (Wu *et al*., 2005; Ruiz-Lopez *et al*., 2012). However, effective accumulation of the target fatty acids, eicosapentaenoic acid (20:5 *n*-3, EPA) and especially docosahexaenoic acid (22:6 *n*-3, DHA), at levels comparable to those in fish has proved technically challenging to date, despite many attempts to optimize their synthesis (see Figure1a for a simplified representation of biosynthetic pathway; Ruiz-Lopez *et al*., 2012; Haslam *et al*., 2013). One advance in increasing the accumulation of omega-3 LC-PUFAs in transgenic plants has derived from characterization of acyl CoA-dependent desaturases (Sayanova *et al*., 2011a), which bypass the well-documented substrate-dichotomy bottleneck (a result of the differing acyl carrier substrate preferences of desaturases and elongases; Abbadi *et al*., 2004). The benefits of using an acyl CoA-dependent Δ6-desaturase, such as that found in *Ostrococcus tauri* (Domergue *et al*., 2005), include not only the possibility of elevated EPA accumulation, but also a route to circumvent unwanted C18 biosynthetic intermediates (Sayanova *et al*., 2011a; Ruiz-Lopez *et al*., 2013). Accumulation of these intermediates (usually omega-6 acids) is characteristically associated with expression of phospholipid-dependent Δ6-desaturases (Abbadi *et al*., 2004; Sayanova *et al*., 2011a; Ruiz-Lopez *et al*., 2013) and is not seen in fish oil. Indeed, previous attempts to produce EPA have resulted in massive co-accumulation of the C18 omega-6 γ-linolenic acid (18:3 *n*-6, GLA) (Cheng *et al*., 2010).

Here, we demonstrate how a crop (*C. sativa*) may be successfully engineered to accumulate high levels of EPA and/or DHA in its seed oil. The choice of *C. sativa* as a host for the biosynthesis of omega-3 LC-PUFAs enables the introduced enzymes to use the endogenously accumulating high levels of α-linolenic acid (18:3 *n*-3, ALA), the starting substrate for the omega-3 biosynthetic pathway (Figure1a). Furthermore, the high yield of target fatty acids was achieved without unwanted accumulation of intermediates and/or omega-6 PUFAs in the seed oil, and, most importantly, with efficient channelling of EPA and DHA into seed triacylglycerols.

## Results and Discussion

### Production of EPA and DHA in an oil seed crop

Two constructs encoding the primary biosynthetic activities for omega-3 LC-PUFA biosynthesis (p5_EPA and p7_DHA) were prepared for introduction into *C. sativa*. Construct p5_EPA was designed to produce high levels of EPA in seeds and contained five genes (Figure1b), while construct p7_DHA was designed to accumulate both EPA and DHA, and contained seven genes (Figure1b). In both cases, individual biosynthetic activities were encoded by codon-optimized sequences under the independent control of seed-specific promoters (Figure1b; see also Experimental procedures). These two constructs were introduced into *C. sativa* plants via *Agrobacterium*-mediated floral transformation. The transformation of *C. sativa* and expression of these constructs resulted in no change in the germination, development or stature of the transgenic plants (Figure S1). Analysis of total fatty acid methyl esters (FAMEs) from the seeds of eight individual transgenic *C*. *sativa* T_2_ lines expressing the p5_EPA construct demonstrated accumulation of significant levels of EPA (Table S1). The mean levels of EPA found in these T_2_ lines ranged from 6.8 to 18.7% of total fatty acids in mature seeds (mean 13.8%). As observed previously, the amount of oleic acid (18:1 *n*-9, OA) decreased dramatically from 14.5% in the untransformed control to 5.9% in transgenic *C. sativa* seeds expressing the *O. tauri* Δ6-desaturase (Sayanova *et al*., 2011a). In agreement with previous results obtained with expression of acyl CoA Δ6-desaturases in transgenic plants (Hoffmann *et al*., 2008; Petrie *et al*., 2010; Sayanova *et al*., 2011a; Ruiz-Lopez *et al*., 2012, 2013), only a minor accumulation of C18 Δ6-desaturated fatty acids was observed: the mean levels of GLA and stearidonic acid (18:4 *n*-3, SDA) in T_2_ seeds were 2.7% and 2.9% of total fatty acids, respectively (Table S1). Similarly, the mean levels of the omega-6 LC-PUFA intermediates arachidonic acid (20:4 *n*-6, ARA) and dihomo-γ-linolenic acid (20:3 *n*-6, DGLA) were 1.7% and 0.8%, respectively. Overall, levels of newly synthesized omega-3 fatty acids were much higher than those of omega-6 acids, with an *n*-3/*n*-6 ratio of 9:1. It is interesting to note that the health-promoting effects of LC-PUFAs are dependent on maintenance of the correct balance between omega-3 and omega-6 PUFA. A lower ratio of omega-6/omega-3 fatty acids is more desirable to reduce the risk of many of the chronic diseases of now prevalent in Western societies.

**Figure 2:**
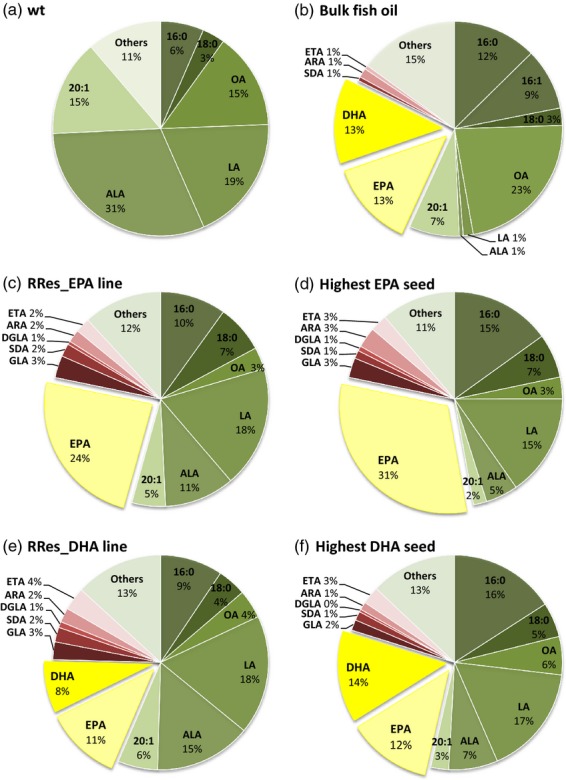
Total fatty acid composition (mol%) of wild-type and engineered *C. sativa* oilseeds. Distribution of FAMEs in (a) wild-type *C. sativa*, (b) bulk fish oil, (c) the RRes_EPA line, (d) the highest EPA seed, (e) the RRes_DHA line and (f) the highest DHA seed. Endogenous fatty acids are shown in shades of green; intermediates of the introduced biosynthetic pathway are shown in shades of red, and the key target fatty acids (EPA and DHA) are shown in shades of yellow.

Analysis of seed FAMEs in control (Figure2a) and T_3_ lines expressing p5_EPA showed an increase in EPA content (14.7% of total fatty acids in seeds). In contrast to the mean T_2_ value, the highest EPA value observed in an individual T_3_ line (named RRes_EPA) was 23.9% (Figure2c and Table S1). Further analyses demonstrated that the highest EPA value observed in an individual seed of RRes_EPA was 31% of total fatty acids (Figure2d). Previous reports on high EPA accumulations (e.g. Cheng *et al*., 2010) focused on the zero-erucic acid *Brassica carinata*, with a mean EPA level of 20.4% in transgenic seeds. However, the potential of *B. carinata* as a source of omega-3 was compromised by the inefficient Δ6-elongation step, which resulted in accumulation of GLA and SDA (26.9% and 5.4% of total fatty acids, respectively).

After achieving a significant yield of EPA, the next goal was to produce both EPA and DHA in *C. sativa* seeds to levels similar to those in fish oil (Figure2b). Engineering a metabolic route to DHA concomitant with EPA has been challenging, with many efforts resulting in some accumulation of EPA and docosapentaenoic acid (22:5 *n*-3; DPA), although characteristically only small DHA accumulations were recorded. We and others have shown how DHA may be accumulated in the seed oil of the model Arabidopsis, with accumulation of up to 15% DHA in Arabidopsis seed oil (Petrie *et al*., 2012; Ruiz-Lopez *et al*., 2013). However, levels of EPA in this line were extremely low (1.8%; Petrie *et al*., 2012). Combining our knowledge of how to produce significant accumulations of EPA with our previous experience of DHA synthesis in Arabidopsis (Ruiz-Lopez *et al*., 2012, 2013), we identified a transgene combination (p7_DHA) to successfully direct accumulation of significant levels of EPA and DHA in *C. sativa*. The mean yields of EPA and DHA in *C. sativa* T_2_ lines expressing p7_DHA were 6.2% and 5.2% of total fatty acids in seeds (Table S1). Moreover, as was the case with RRes_EPA, these lines showed low accumulation of C18 Δ6-desaturated fatty acids and ARA. The line accumulating the highest amount of DHA (named RRes_DHA) showed a mean accumulation of 11% EPA and 8% DHA in its seeds (Figure2e). In addition, further single-seed analysis of the RRes_DHA line revealed individual seeds that accumulated up to 12% EPA and 14% DHA (Figure2f), values very similar to those found in fish oil (mean of 13% EPA and 13% DHA, Figure2b), but completely absent from wild-type *C. sativa* (Figure2a). The accumulation of EPA and DHA was achieved with only minimal synthesis of intermediates (Figure2, shown in shades of red), thus avoiding problems in human fatty acid metabolism or interference with the benefits of omega-3 fatty acids.

**Figure 3:**
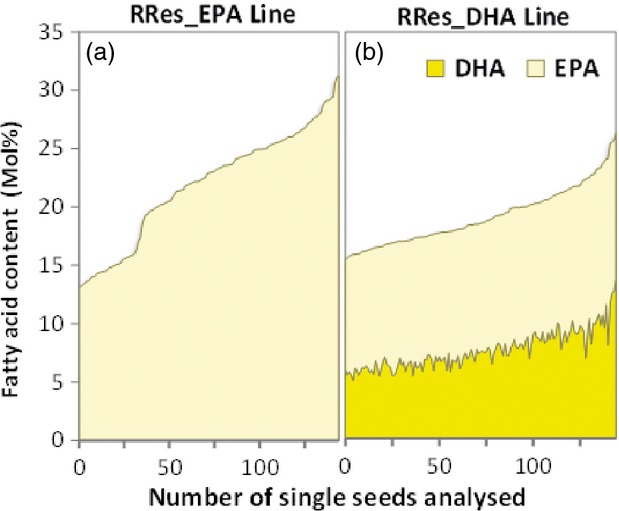
Distribution of EPA and/or DHA content (mol% of total fatty acids) in approximately 145 single seeds of RRes_EPA and RRes_DHA lines. FAMEs of single seeds derived from selected T_2_ events for either RRes_EPA (*n *=* *8) or RREs_DHA (*n *=* *12) were analysed by GC-FID (flame ionization detection), and the mol% for either EPA (a) or EPA and DHA (b) is shown. See also Table S1.

Importantly, and distinct from the observations of others in Arabidopsis (Petrie *et al*., 2011, 2012), significant levels of EPA and DHA were present in a high proportion of the individual seeds analysed from selected T_2_ lines (Figure3a,b and Table S1). This is a clear demonstration of the potential of engineered *C. sativa* seeds as a source of EPA and DHA, and represents a breakthrough in identification of sustainable alternative sources for these target fatty acids.

### Lipid profiling of omega-3-accumulating lines

To further characterize the accumulation of LC-PUFAs in transgenic *C. sativa*, a more detailed lipid class analysis was performed on seeds of both RRes_EPA and RRes_DHA lines. Analyses of neutral and polar lipids from the seed oil of RRes_EPA revealed that EPA was almost equally distributed between neutral lipids (19%) and polar lipids (17.1%; see Table1). This pattern of partitioning is slightly reversed compared with our previous observations in Arabidopsis seeds expressing a similar EPA construct, where EPA comprised 8.7% of total fatty acids in neutral lipids and 11.5% in polar lipids (Ruiz-Lopez *et al*., 2013). Characterization of seed lipids from RRes_DHA clearly showed accumulation of DHA in neutral lipids, although polar lipids showed a higher accumulation of DHA (8.5% versus 4.5%; Table1).

**Table 1:** Fatty acid composition (mol%; *n *=* *3, means ± SD) of total lipids, neutral lipids and polar lipids isolated from seeds of wild-type *C. sativa* (WT; Col-0), and transgenic *C. sativa* lines producing EPA and DHA DPA = docosapentaenoic acid

	16:0	18:0	OA	LA	GLA	ALA	SDA	20:1	DGLA	ARA	ETA	EPA	DPA	DHA	Others
Total lipids															
WT	6.5 ± 0.1	3.4 ± 0.1	14.5 ± 0.4	19.0 ± 0.6	–	30.8 ± 0.6	–	14.5 ± 0.1	–	–	–	–	–	–	11.2 ± 0.1
RRes_EPA	9.3 ± 0.5	5.8 ± 0.3	3.3 ± 0.4	18.3 ± 0.2	1.6 ± 0.3	13.4 ± 2.6	1.0 ± 0.2	5.6 ± 0.7	1.1 ± 0.3	2.0 ± 0.1	4.1 ± 0.6	23.1 ± 2.6	1.1 ± 0.0	–	10.3 ± 0.4
RRes_DHA	9.5 ± 0.1	5.0 ± 0.1	5.8 ± 0.3	19.2 ± 0.3	4.1 ± 0.2	11.7 ± 0.7	3.4 ± 0.1	7.2 ± 0.2	0.8 ± 0.0	2.4 ± 0.1	2.4 ± 0.1	10.7 ± 0.2	2.4 ± 0.1	6.2 ± 0.1	9.1 ± 0.3
Neutral lipids															
WT	7.1 ± 0.1	3.2 ± 0.0	15.3 ± 0.1	18.9 ± 0.2	–	31.8 ± 0.3	–	15.3 ± 0.1	–	–	–	–	–	–	8.4 ± 0.0
RRes_EPA	8.1 ± 0.1	7.4 ± 0.1	5.2 ± 0.1	19.4 ± 0.1	1.6 ± 0.1	13.1 ± 0.2	0.9 ± 0.1	8.1 ± 0.1	1.2 ± 0.1	1.9 ± 0.0	3.6 ± 0.1	19.0 ± 0.6	1.0 ± 0.0	–	9.5 ± 0.1
RRes_DHA	8.2 ± 0.0	5.1 ± 0.1	7.8 ± 0.1	21.5 ± 0.1	5.1 ± 0.3	14.4 ± 0.1	4.6 ± 0.4	8.5 ± 0.1	0.9 ± 0.1	1.9 ± 0.1	2.2 ± 0.1	7.4 ± 0.4	1.5 ± 0.1	4.5 ± 0.2	6.3 ± 0.1
Polar lipids															
WT	14.0 ± 0.8	4.4 ± 0.4	35.0 ± 0.9	27.7 ± 0.4	–	12.8 ± 0.5	–	3.2 ± 0.2	–	–	–	–	–	–	3.0 ± 0.3
RRes_EPA	18.8 ± 0.4	9.8 ± 0.2	5.3 ± 0.2	22.3 ± 0.8	1.3 ± 0.1	6.9 ± 0.3	0.5 ± 0.1	4.3 ± 0.1	1.1 ± 0.0	1.4 ± 0.0	3.5 ± 0.1	17.1 ± 0.3	2.1 ± 0.2	–	5.7 ± 0.1
RRes_DHA	18.4 ± 0.2	6.3 ± 0.2	7.5 ± 1.2	21.7 ± 0.7	5.3 ± 0.4	7.2 ± 0.5	2.2 ± 0.2	3.9 ± 0.2	1.1 ± 0.1	1.0 ± 0.1	2.4 ± 0.2	5.9 ± 0.5	5.2 ± 0.7	8.5 ± 1.0	3.3 ± 0.1
Glycolipids															
WT	16.9 ± 1.5	8.8 ± 1.4	14.0 ± 0.7	15.0 ± 1.4	–	21.8 ± 2.7	–	9.5 ± 0.3	–	–	–	–	–	–	14.0 ± 2.5
RRes_EPA	21.5 ± 0.9	15.6 ± 1.3	4.3 ± 0.3	16.6 ± 1.0	1.1 ± 0.1	6.9 ± 0.8	0.4 ± 0.1	8.8 ± 0.4	0.9 ± 0.1	1.2 ± 0.2	1.8 ± 0.3	9.0 ± 1.6	1.7 ± 0.8	–	10.2 ± 0.6
RRes_DHA	20.6 ± 1.0	13.1 ± 0.8	3.4 ± 0.9	16.3 ± 0.6	3.8 ± 0.2	11.0 ± 0.4	2.3 ± 0.1	4.4 ± 0.8	0.6 ± 0.0	1.6 ± 0.2	2.2 ± 0.7	5.5 ± 0.5	2.4 ± 0.9	6.3 ± 0.6	6.4 ± 0.8

To better understand the distribution of these non-native fatty acids in *C. sativa* seeds, we performed a more detailed examination of the lipidome, using similar approaches as previously described for Arabidopsis (Ruiz-Lopez *et al*., 2013). First, the distribution of EPA and DHA in neutral lipids was determined for the RRes_EPA and RRes_DHA lines, compared with wild-type *C. sativa* (Table2). Interestingly, this revealed a difference in the accumulation of EPA and DHA into triacylglycerols (TAG), as EPA levels were greater in TAG than diacylglycerols (DAG) for RRes_EPA, whereas the reverse was true for DHA in RRes_DHA. To develop a more accurate hypothesis regarding the flux of these fatty acids through various lipid classes, we also determined the acyl composition of the major phospholipids phosphatidylcholine (PC), phosphatidylethanolaomine (PE), phosphatidylinositol (PI) and phosphatidylserine (PS) (Table3 and Figure S2A–F) in order to evaluate the PC-DAG-TAG model proposed by Bates and Browse (2012), in which extraplastidial fatty acids accumulate in triacylglycerol via PC and then DAG, rather than direct acylation into neutral lipids from the acyl CoA pool. The fatty acid composition of PC, PE and PI+PS was determined for RRes_EPA, RRes_DHA and wild-type *C. sativa* mature seeds (Table3 and Figure S2A–F), revealing a precise pattern of distribution for non-native omega-3 LC-PUFAs among these phospholipids. In the case of EPA in RRes_EPA, there was a very similar level of accumulation (approximately 15%) in all phospholipids, whereas in RRes_DHA, the accumulation of DHA was slightly biased towards PE, with little accumulation in PI+PS (Table3). Such a pattern of accumulation of EPA and DHA is similar to that observed in transgenic Arabidopsis seeds accumulating these fatty acids (Ruiz-Lopez *et al*., 2013). Examination of the accumulation of EPA and DHA across PC, DAG and TAG (Tables2 and 3) revealed discrete patterns for retention or accumulation of these two fatty acids. In the case of EPA, accumulation follows the pattern TAG > DAG > PC (21.1%; 17.8%: 15.6%; Tables2 and 3), consistent with the Bates and Browse (2012) model of flux through PC. Similar patterns of accumulation are observed for endogenous fatty acids such as ALA and 20:1. In the case of DHA, the accumulation pattern is reversed to PC > DAG > TAG (7.3%; 5.9%; 4.5%; Tables2 and 3), indicating inefficient flux of this C22 LC-PUFA compared with the C20 form. Similar PC-biased accumulation of DHA was previously observed in Arabidopsis (Ruiz-Lopez *et al*., 2013), although a similar pattern was also observed for EPA in that species. However, in the present case of *C. sativa*, it is clear that, while EPA accumulates in TAG via DAG (presumably derived from PC, rather than via *de novo* synthesis, according to the model proposed by Bates and Browse, 2012), this is not the case for DHA. Clearly, TAG synthesis is an aggregation of a number of distinct pathways, and, in transgenic *C. sativa*, there appears to also be some acyl exchange between the glycerolipid pool and the acyl CoA pool, as both EPA and DHA are present in the latter (Table4). In the case of DHA, this may represent an alternative route into TAG.

**Table 2:** Fatty acid composition (mol%; *n *=* *3, means ± SD) of neutral lipids isolated from seeds of wild-type *C. sativa* (WT; Col-0), and transgenic *C. sativa* lines producing EPA and DHA

	16:0	18:0	OA	LA	GLA	ALA	SDA	20:1	DGLA	ARA	ETA	EPA	DPA	DHA	Others
Neutral lipids															
WT	7.1 ± 0.1	3.2 ± 0.0	15.3 ± 0.1	18.9 ± 0.2	–	31.8 ± 0.3	–	15.3 ± 0.1	–	–	–	–	–	–	8.4 ± 0.0
RRes_EPA	8.1 ± 0.1	7.4 ± 0.1	5.2 ± 0.1	19.4 ± 0.1	1.6 ± 0.1	13.1 ± 0.2	0.9 ± 0.1	8.1 ± 0.1	1.2 ± 0.1	1.9 ± 0.0	3.6 ± 0.1	19.0 ± 0.6	1.0 ± 0.0	–	9.5 ± 0.1
RRes_DHA	8.2 ± 0.0	5.1 ± 0.1	7.8 ± 0.1	21.5 ± 0.1	5.1 ± 0.3	14.4 ± 0.1	4.6 ± 0.4	8.5 ± 0.1	0.9 ± 0.1	1.9 ± 0.1	2.2 ± 0.1	7.4 ± 0.4	1.5 ± 0.1	4.5 ± 0.2	6.3 ± 0.1
TAG															
WT	7.7 ± 0.1	3.4 ± 0.0	13.8 ± 0.4	18.6 ± 0.4	–	32.9 ± 0.6	–	15.1 ± 0.4	–	–	–	–	–	–	8.6 ± 0.1
RRes_EPA	8.1 ± 0.9	6.9 ± 0.6	4.8 ± 0.4	18.0 ± 0.3	1.7 ± 0.1	13.5 ± 1.0	0.9 ± 0.0	7.8 ± 0.4	1.2 ± 0.1	2.1 ± 0.1	3.8 ± 0.3	21.2 ± 1.2	1.1 ± 0.1	–	9.1 ± 0.5
RRes_DHA	8.8 ± 0.2	5.3 ± 0.4	6.9 ± 0.5	20.3 ± 0.3	5.3 ± 0.5	14.2 ± 0.3	4.5 ± 0.6	8.0 ± 0.4	0.9 ± 0.1	2.1 ± 0.1	2.4 ± 0.2	8.4 ± 1.0	1.5 ± 0.1	4.9 ± 0.3	6.4 ± 0.3
DAG															
WT	11.8 ± 0.4	8.3 ± 0.0	19.5 ± 0.9	16.1 ± 0.1	–	21.0 ± 0.1	–	10.7 ± 0.5	–	–	–	–	–	–	12.5 ± 0.1
RRes_EPA	13.3 ± 0.4	10.5 ± 0.2	4.9 ± 2.0	17.3 ± 0.6	1.5 ± 0.1	9.1 ± 0.3	0.8 ± 0.1	5.7 ± 0.4	1.0 ± 0.0	1.8 ± 0.0	3.6 ± 0.3	17.8 ± 0.9	1.2 ± 0.0	–	11.3 ± 0.9
RRes_DHA	10.7 ± 0.5	7.2 ± 0.3	7.9 ± 1.0	19.6 ± 0.7	4.4 ± 0.2	10.9 ± 0.7	3.6 ± 0.2	6.2 ± 0.4	0.8 ± 0.0	1.9 ± 0.2	2.4 ± 0.6	8.5 ± 0.3	1.9 ± 0.2	5.9 ± 0.3	8.0 ± 0.7

**Table 3:** The distribution and content of polar lipids isolated from seeds of wild-type (WT; Col-0) and transgenic *C. sativa* lines producing EPA and DHA (mol%; *n *=* *3, means ± SD)

	Fatty acid composition (mol%)														
16:0	18:0	OA	LA	GLA	ALA	SDA	20:1	DGLA	ARA	ETA	EPA	DPA	DHA	Others
Total polar lipids															
Wild-type	14.0 ± 0.8	4.4 ± 0.4	35.0 ± 0.9	27.7 ± 0.4	–	12.8 ± 0.5	–	3.2 ± 0.2	–	–	–	–	–	–	3.0 ± 0.3
RRes_EPA	18.8 ± 0.4	9.8 ± 0.2	5.3 ± 0.2	22.3 ± 0.8	1.3 ± 0.1	6.9 ± 0.3	0.5 ± 0.1	4.3 ± 0.1	1.1 ± 0.0	1.4 ± 0.0	3.5 ± 0.1	17.1 ± 0.3	2.1 ± 0.2	–	5.7 ± 0.1
RRes_DHA	18.4 ± 0.2	6.3 ± 0.2	7.5 ± 1.2	21.7 ± 0.7	5.3 ± 0.4	7.2 ± 0.5	2.2 ± 0.2	3.9 ± 0.2	1.1 ± 0.1	1.0 ± 0.1	2.4 ± 0.2	5.9 ± 0.5	5.2 ± 0.7	8.5 ± 1.0	3.3 ± 0.1
PC															
Wild-type	14.1 ± 1.3	7.3 ± 1.7	39.0 ± 2.1	24.3 ± 1.0	–	10.5 ± 0.4	–	1.7 ± 0.1	–	–	–	–	–	–	2.0 ± 0.4
RRes_EPA	16.3 ± 1.0	10.6 ± 1.1	1.8 ± 0.0	24.7 ± 0.9	1.3 ± 0.1	6.6 ± 0.7	1.1 ± 0.2	4.7 ± 0.4	1.3 ± 0.0	1.2 ± 0.0	3.5 ± 0.5	15.6 ± 2.0	2.2 ± 0.1	–	9.2 ± 1.8
RRes_DHA	15.9 ± 1.6	6.5 ± 0.3	4.1 ± 1.8	19.9 ± 1.2	5.3 ± 0.5	6.8 ± 0.6	3.6 ± 0.5	5.8 ± 0.8	1.7 ± 0.1	1.2 ± 0.1	2.7 ± 0.1	6.1 ± 0.5	5.4 ± 0.5	7.3 ± 0.9	7.8 ± 1.4
PE															
Wild-type	14.9 ± 1.2	2.1 ± 0.3	29.0 ± 0.7	34.6 ± 1.4	–	13.5 ± 0.6	–	1.0 ± 0.1	–	–	–	–	–	–	4.4 ± 1.2
RRes_EPA	18.1 ± 1.8	10.5 ± 0.5	2.5 ± 0.7	23.0 ± 0.9	1.7 ± 0.2	5.9 ± 0.6	1.5 ± 0.2	3.5 ± 0.6	1.0 ± 0.2	1.4 ± 0.2	3.0 ± 0.5	15.3 ± 2.1	2.3 ± 0.3	–	10.4 ± 0.3
RRes_DHA	18.3 ± 0.2	5.8 ± 0.3	1.9 ± 0.3	20.2 ± 1.9	5.8 ± 0.5	4.1 ± 0.3	3.0 ± 0.1	3.6 ± 0.5	0.9 ± 0.0	0.5 ± 0.2	1.6 ± 0.2	4.9 ± 0.7	4.3 ± 0.2	9.1 ± 0.6	16.1 ± 0.9
PI & PS															
Wild-type	16.0 ± 1.1	8.1 ± 0.3	24.3 ± 0.2	25.2 ± 0.2	–	17.7 ± 0.0	–	1.1 ± 0.1	–	–	–	–	–	–	6.7 ± 0.8
RRes_EPA	24.8 ± 2.1	15.2 ± 1.5	2.5 ± 1.2	14.8 ± 2.0	3.1 ± 0.6	6.4 ± 0.5	2.3 ± 0.5	3.8 ± 0.8	1.1 ± 0.2	0.9 ± 0.1	1.5 ± 0.1	14.1 ± 3.5	1.3 ± 0.2	–	8.1 ± 2.3
RRes_DHA	27.4 ± 4.0	12.0 ± 1.4	4.9 ± 1.9	16.0 ± 2.3	3.2 ± 0.2	6.2 ± 0.3	3.0 ± 0.4	3.9 ± 1.0	0.5 ± 0.6	1.0 ± 0.4	0.9 ± 0.1	7.8 ± 1.5	2.1 ± 0.6	2.7 ± 0.6	8.3 ± 0.9

**Table 4:** Acyl CoA composition of *C. sativa* seed

Acyl CoA	Wild-type	RRes_EPA	RRes_DHA
16:0	12.8 ± 3.0	8.9 ± 0.7	8.6 ± 0.7
18:0	20.2 ± 1.9	16.4 ± 4.1	17.1 ± 1.3
18:1	6.5 ± 0.9	2.0 ± 0.3	2.6 ± 0.7
18:2	15.0 ± 5.3	3.2 ± 0.7	3.1 ± 0.5
18:3	4.5 ± 1.2	4.4 ± 0.9	4.2 ± 0.8
SDA	0.0	1.5 ± 0.7	1.4 ± 0.6
20:0	19.1 ± 4.9	23.4 ± 0.7	22.6 ± 0.6
20:1	5.9 ± 0.4	5.5 ± 0.8	5.3 ± 0.5
20:3	0.2 ± 0.1	2.7 ± 0.3	2.6 ± 0.2
20:4	0.0	3.8 ± 0.5	3.6 ± 0.4
EPA	0.0	8.3 ± 1.6	4.2 ± 0.8
22:0	6.4 ± 2.2	8.6 ± 0.5	8.3 ± 0.5
22:1	2.1 ± 0.9	1.8 ± 0.2	1.8 ± 0.1
DPA	0.0	0.0	1.7 ± 0.9
DHA	0.0	0.0	3.9 ± 0.9

The acyl CoA species were determined by LC-MS/MS + MRM. Values are means ± SD for seed from four plants of each genotype at 28 DAF.

As well as the accumulation patterns for EPA and DHA described above, there are a number of other alterations to the lipid composition of transgenic *C. sativa* accumulating EPA and DHA, the most notable of which is a very pronounced decrease in the accumulation of oleic acid (OA; 18:1^Δ9^). This is true for both EPA- and DHA-accumulating lines, and was observed in neutral lipids (Table2), phospholipids (Table3) and acyl CoAs (Table4). One interpretation for this inverse relationship with omega-3 LC-PUFA accumulation is that OA acts as a primary substrate for the synthesis of EPA and DHA, with distinct pools of this fatty acid (most likely in DAG) being channelled towards their synthesis. The fact that accumulation of endogenous metabolites of OA such as linoleic acid (LA; 18:2^Δ912^) (desaturation) or 20:1 (elongation) is not so dramatically altered in the transgenic lines compared with the wild-type may be interpreted as supporting such a scenario. Interestingly, and again highlighting the difference between *C. sativa* and Arabidopsis, the most dramatically alteration in fatty acid level in transgenic omega-3 LC-PUFA-accumulating Arabidopsis lines was a decrease in LA, which was observed in both neutral and phospholipid fractions (Ruiz-Lopez *et al*., 2013). Thus, while our data suggest the importance of a PC-derived DAG pool in TAG synthesis in *C. sativa*, analogous to Arabidopsis, they also suggest some significant features that make *C. sativa* a superior host for accumulation of EPA and DHA, and also question the assertion that Arabidopsis is a useful predictive model for events in *C. sativa*. However, further studies are required to better address this latter point.

**Figure 4:**
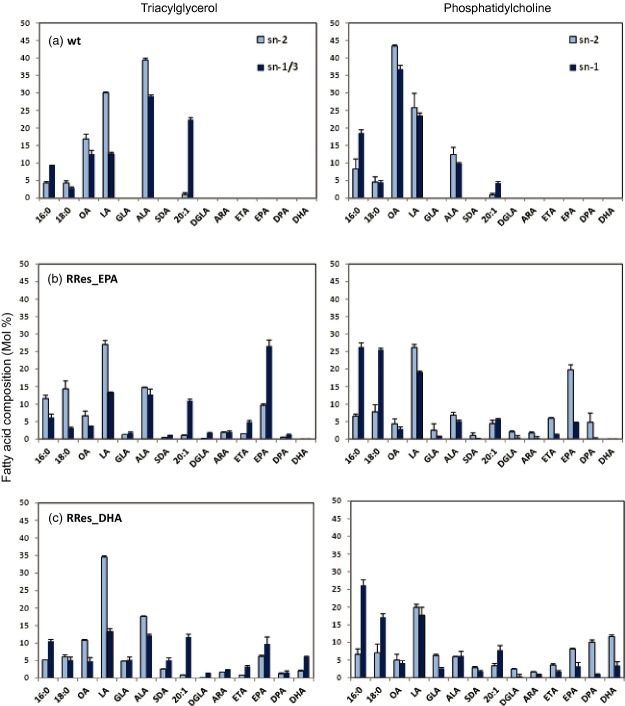
Stereospecific analysis of PC and TAG in RRes_EPA and RRes_DHA lines. The positional distribution of fatty acids in TAG (*sn*-2 and *sn*-1/3) and PC (*sn*-1 and *sn*-2) was determined in wild-type *C. sativa* (a), RRes_EPA (b) and RRes_DHA (c).

To further define the acyl acylation of EPA and DHA into seed oil, and also possible biosynthetic fluxes, region-specific analyses were performed on RRes_EPA and RRes_DHA in order to determine the position on the glycerol backbone of EPA and DHA in both TAG and PC molecules. This analysis demonstrated the inherent bias of *C. sativa* acyltransferases with regard to positioning EPA and DHA at the *sn*-1/*sn*-3 positions in seed TAG (Figure4). This was also true for eicosatetraenoic acid (20:4 n-3; ETA) and endogenous 20:1, whereas some endogenous fatty acids (such as OA, LA and to a lesser extent ALA) were biased towards the *sn*-2 position of TAG (Figure4). The positional distribution of EPA and DHA showed a threefold enrichment at the *sn*-1/*sn*-3 position compared with the *sn*-2 position. Similarly, the distribution of EPA and DHA in PC was biased towards the *sn*-2 position, confirming the critical role of *sn*-2 PC in the biosynthesis (Δ5 and Δ4-desaturation) of omega-3 LC-PUFAs. Therefore, EPA and DHA are ultimately synthesized on *sn*-2 PC, and then channelled from this lipid to the *sn*-3 position of TAG, although whether this occurs directly via acyl CoA-independent pathways or via acyl exchange into the acyl CoA pool and subsequent acyl CoA-dependent acylation is unclear. As discussed above, the role of DAG as an intermediate may be inferred, at least in the case of EPA, although it remains to be determined if this occurs by incorporation into *de novo* DAG or via head group exchange with PC.

However, our studies confirm the crucial role that the endogenous seed lipid metabolism of the host plant plays in successful reconstitution of this heterologous pathway. Given that this varies from species to species, a clear understanding of the biochemical context into which transgenic metabolic pathways are inserted is essential, as recommended by Bates and Browse (2012) in their discussion of the accumulation of unusual C18 fatty acids.

### Characterization of omega-3 in TAG molecular species

To better understand the accumulation of the target fatty acids within TAG, we performed LC-MS/MS analyses to identify the individual molecular species of TAG containing EPA and/or DHA present in mature seed oils of transgenic RRes_EPA and RRes_DHA T3 lines. As a result, a diverse collection of EPA- and DHA-containing TAG was identified (Figure S3A–C). In the RRes_EPA line (Figure S3A), the predominant EPA-containing TAG comprised TAG species containing a single EPA chain. The most abundant TAG species were EPA–OA–LA, EPA–ALA–20:1, EPA–18:0–20:1 and EPA–20:0–20:3, with mean levels of 11.5%, 10.0%, 11.0% and 8.7% of total EPA-containing TAG, respectively. Four TAG species in the RRes_EPA line comprised two EPA and one of the following fatty acids: LA, 20:0, 20:1 or ARA (13.0% of total EPA TAG). We also detected a tri-EPA TAG (1.5% of total EPA TAG). The capacity of *C. sativa* TAG to accommodate incorporation of more EPA (and DHA) was examined by parallel FAME analyses of each sample (Figure S4A); the data clearly demonstrate how the contribution of TAG species containing two or more EPA increased proportionately with the percentage total EPA content. Furthermore, parallel FAME analysis of RRes_DHA TAG (Figure S4B) showed that concomitant accumulation of EPA and DHA is not restricted by TAG biosynthesis in *C. sativa* seed oil.

Targeted TAG LC-MS/MS analysis of the RRes_DHA line (Figure S3B,C) identified the major DHA-containing TAG. These were DHA–LA–ALA (mean 14.5%), DHA–LA–LA (13.8%) and DHA–ALA–ALA (11.3%), which are notably different in their composition of endogenous fatty acids compared with TAG from RRes_EPA (above). We also observed appreciable levels of TAG containing two DHA molecules (6.8% of total DHA-containing TAG) and very low levels of tri-DHA TAG (approximately 0.3% of total DHA TAG). Both EPA and DHA were also found in the same TAG species (Figure S5), e.g. in combination with OA and 18:0. There was also a modification of the pattern of EPA accumulation in the TAG species of the RRes_DHA line. In order to accommodate the synthesis and storage of DHA, single molecules of EPA were distributed across a wider variety of TAG species, rather than accumulating in the four specific TAG seen in RRes_EPA. Collectively, these observations on the accumulation of EPA or DHA confirm the disparity in the channelling of these two non-native fatty acids, and indicate the need for further studies to better define their path of deposition in TAG.

Different plants probably utilize a combination of routes to incorporate novel fatty acids into TAG (Bates *et al*., 2013). However, it is clear that PC plays a central role, with newly synthesized LC-PUFAs, such as EPA and DHA, under a constant dynamic exchange with the acyl CoA pool in a process described as acyl editing. Removal of an LC-PUFA from PC may proceed by the reverse action of acyl CoA:lysophosphatidylcholine acyltransferase or the combined action of phospholipase A2 and long-chain acyl CoA synthetase. Once in the acyl CoA pool, EPA-CoA and DHA-CoA and glycerol-3-phosphate may be converted into TAG in a series of reactions known as the Kennedy pathway (Bates and Browse, 2012). Alternatively, the PC head group may be removed, producing a DAG molecule containing EPA or DHA at the *sn*-2 position. This reaction may proceed via four enzymatic mechanisms: phospholipase C, phospholipase D together with phosphatidic acid phosphatase, the reverse action of CDP-choline:diacylglycerol cholinephosphotransferase, or the recently identified phosphatidylcholine:diacyglycerol cholinephosphotransferase. The DAG produced by these mechanisms may then be utilized to produce TAG. Furthermore, direct transfer of the *sn*-2 EPA or DHA of PC to the *sn*-3 position of DAG is possible, generating TAG via a phospholipid:diacylglycerol acyltransferase. The analysis of *C. sativa* seed oil has demonstrated the capacity of this plant to accumulate high levels of novel fatty acids in TAG, and suggests that there may be specific lipid pools, separate to those for membrane synthesis, that channel EPA and/or DHA into TAG. How this may be achieved remains unclear: it may involve a subset of specific enzymes or a spatial separation in the ER membrane (see Chapman and Ohlrogge, 2012); however, it is clear that manipulation of plant seed oil composition is heavily dependent on the metabolic pathways of the host.

In conclusion, the accumulation of high levels of EPA and/or DHA in plant oils is an important goal of the metabolic engineering community. In this study, we have successfully reconstituted the EPA and DHA biosynthetic pathway in the seeds of an oilseed crop, *C. sativa*, demonstrating not only the practical feasibility of large-scale production of these important omega-3 fatty acids in an oilseed crop at comparable levels to those found in fish oils, but also a composition that matches that of marine sources.

## Experimental Procedures

### Plant material and growth conditions

*Camelina sativa* was grown for analyses in a controlled-environment chamber at 23°C day/18°C night, 50–60% humidity, and kept under a 16 h photoperiod (long day) at 250 μmol m^−2 ^sec^−1^.

### Generation of transgenic plants

Transgenic *C. sativa* lines were generated as previously described with minor modifications (Lu and Kang, 2008; Nguyen *et al*., 2013). The designed vectors were transferred into *Agrobacterium tumefacians* strain AGL1. *C. sativa* inflorescences were immersed in the *Agrobacterium* suspension for 30 sec without applying any vacuum. Transgenic seeds expressing the DHA pathway were identified by visual screening for DsRed activity.

Seeds harvested from transformed plants were illuminated using a green LED light. Fluorescent seeds were visualized using a red lens filter. In all cases, no phenotypic perturbation was observed as a result of modification of the seed oil composition.

### Vector construction

Two constructs containing cassettes of five or seven genes were used for plant transformation. The five-gene construct, p5_EPA (Figure1b), contained an optimal set of genes for EPA synthesis: a Δ6-desaturase gene from *O. tauri* (OtΔ6; Domergue *et al*., 2005), a Δ6 fatty acid elongase gene from *Physcomitrella patens* (PSE1, Zank *et al*., 2002) a Δ5-desaturase gene from *Thraustochytrium* sp. (TcΔ5; Qiu *et al*., 2001), a Δ12-desaturase gene from *Phytophthora sojae* (PsΔ12; Bauer *et al*., 2012) to enhance the levels of LA CoA (as substrate for the OtΔ6 enzyme) and an ω3-desaturase from *Phytophthora infestans* (Pi-ω3; Wu *et al*., 2005) to increase the conversion of ARA to EPA. All genes were individually cloned under the control of seed-specific promoters, and then combined into a single T-DNA transformation vector as previously described (Ruiz-Lopez *et al*., 2013). The destination vector contained an *NPT*II gene with the *nos* promoter as a selection marker. All open reading frames for desaturases and elongases were re-synthesized (GenScript Corporation, www.genscript.com) and codon-optimized for expression in *C. sativa*.

To create the seven-gene construct p7_DHA (Figure1b), OtElo5, an *O. tauri* Δ5 fatty acid elongase gene (Meyer *et al*., 2004), and EhΔ4, a Δ4-desaturase gene from *Emiliania huxleyi* (Sayanova *et al*., 2011b), both flanked by conlinin promoters and OCS terminators, were added to the p5_EPA construct. The destination vector contained a DsRed marker for visual selection via seed coat-specific expression of DsRed.

### Fatty acid analysis

Total fatty acids in seed batches were extracted and methylated (Garcés and Mancha, 1993). Methyl ester derivatives of total fatty acids extracted were analysed by GC-FID (flame ionization detection) and the results were confirmed by GC-MS. Values presented are representative numbers derived from replicated analyses.

### Lipid extraction and separation

Three hundred milligrams of seeds were heated for 10 min at 95°C in 1 ml isopropanol, and homogenized using a mortar and pestle. The homogenate was centrifuged at 300 ***g*** for 15 min at room temperature, supernatant was collected, and the pellet was re-extracted with isopropanol/chloroform (1:1 v/v). Both extracts were pooled, evaporated, and dissolved in chloroform/acetic acid (100:1 v/v). The lipid extract was loaded on a Sep-pak column (www.waters.com) and pre-fractionated into neutral lipids, glycolipids and phospholipids by adding chloroform/acetic acid (100:1 v/v), acetone/acetic acid (100:1 v/v) and methanol, respectively. These fractions were further resolved on TLC silica gel plates (thickness 0.25 mm). Neutral lipids were developed using hexane/ethyl ether/formic acid (75:25:1 by volume), and polar lipids were developed using chloroform/methanol/ammonia/water (70:30:4:1 by volume). The individual lipid classes were identified under UV light after spraying with primuline (0.05% w/v in acetone/water, 80:20 v/v), scraped from the plate, and used directly for methylation or extracted for further analysis.

### Positional analysis of TAG and PC

Positional analysis of purified TAG was performed as described previously by Luddy *et al*. (1964). Samples containing 5 mg TAG were dried under nitrogen and re-suspended in 1 ml of 1 mm Tris/HCl (pH 8.0). Samples were then sonicated for 60 sec to ensure complete emulsification of the lipid. Then 0.1 ml of 22% CaCl_2_ and 0.25 ml of 0.1% deoxycolate were added. Samples were warmed at 40°C for 30 sec, and 2 mg pancreatic lipase (≥20 000 units per mg protein, Sigma, www.sigmaaldrich.com) were added. Samples were vortexed for 2–3 min. The reaction was terminated using 0.5 ml 6 m HCl. The lipids were extracted twice with 2.5 ml diethyl ether. Lipids were evaporated at 40°C under nitrogen, and separated into lipid classes by TLC using silica plates and hexane/diethyl ether/acetic acid (70:30:1 by volume). The spots corresponding to 2-monoacylglycerols were scraped from the plate and directly transmethylated for GC-FID analysis. The mean composition of fatty acids in the *sn*-1/3 positions was calculated using the composition of an aliquot of the initial triacylglycerol and the formula: mean percentage *sn*-1/3 =  [(3 x% fatty acid in triacylglycerol) – (% fatty acid in the *sn-*2 position)]/2.

Positional analysis of purified PC was performed as described previously (Ruiz-Lopez *et al*., 2009). Briefly, analysis of PC was performed using *Naja mossambica* phospholipase A2 (Sigma). Samples containing PC were dried under nitrogen and re-suspended in 1 ml borate buffer (0.5 m, pH 7.5, containing 0.4 mm CaCl_2_) by sonication. Five units of lipase and 2 ml diethyl ether were added, and the digestions were performed for 2 h. The ether phase was evaporated, and the reaction was stopped by adding 0.3 ml of 1 m HCl. The aqueous phase was extracted using chloroform/methanol (2:1 v/v). The resulting organic phase was dried under argon and separated by TLC using chloroform/methanol/aqueous ammonia (65:25:0.7 v/v/v) as the solvent mix. The spots corresponding to free fatty acids and lysophospholipids were scraped from the plate, and directly transmethylated for GC-FID analysis.

### Polar lipid analyses

Lipids were extracted from *C. sativa* seed and analysed by ESI-MS/MS using methods adapted from Lee *et al*. (2011). To extract lipids from mature seed, five seeds were crushed, transferred immediately to 3 ml hot isopropanol, and extracted as described above. The extract was sequentially washed for 5 min at room temperature with 1 ml of 1 m KCl and then 2 ml water. The solvent was evaporated under nitrogen, and the dry lipid extract was dissolved in 1 ml chloroform. The molecular species of polar lipids were analysed by ESI triple quadrupole mass spectrometry (API-4000; Applied Biosystems, www.lifetechnologies.com). The molecular species of polar lipid were defined on the basis of the presence of a head-group fragment and the mass/charge of the intact lipid ion formed by ESI. However, tandem ESI-MS/MS precursor and product ion scanning, based on head group fragment, do not determine the individual fatty acyl species. Instead, polar lipids are identified at the level of class, total acyl carbons and total number of acyl carbon–carbon double bonds. Polar lipids were normalized by comparison to a series of polar lipid internal standards, and expressed as a total percentage of the MS peak area signal.

### TAG profiling

Single seeds were extracted in a 1 ml mixture of 33.3% 0.17 m NaCl in methanol, 66.6% heptane and 0.01% butylated hydroxytoluene by volume at 80°C for 2 h as described by Ruiz-Lopez *et al*. (2003). After cooling, the upper heptane TAG phase was transferred to a tapered 1 ml vial (Chromacol, www.chromacol.com) and dried under a stream of nitrogen. The extracted TAG were then re-suspended in 100 μl solvent A (methanol, 0.01% ammonium formate, 0.004% formic acid); 5 μl of this solution were then used for analysis by LC-MS/MS (Agilent 1200, www.agilent.com and ABSciex 4000 QTRAP, www.absciex.com). The TAG species were separated on a Phenomenex (www.phenomenex.com) C18 Kinetex column (2.6 μm, 100 × 2.1 mm) maintained at 40°C, using a gradient of 3% solvent B (100% chloroform) to 20% solvent B over 15 min, then a 4 min gradient to 80% solvent B, holding for 2 min before returning to starting conditions (3% solvent B) for 3 min. Individual TAG species containing EPA and DHA were monitored using multi-reaction monitoring (MRM), tracking the loss of each fatty acid, e.g. TAG 56:7 containing DHA: loss of 16:0 Q1 922.8 m/z – Q3 649.8 m/z, loss of 18:1 Q1 922.8 m/z – Q3 623.8 m/z, loss of 22:6 Q1 922.8 m/z – Q3 577.8 m/z. The internal TAG standards tripentadecanoin (45:0), triheneicosanoin (63:0) and tritricosanoin (69:0) (supplied by Nu-Chek Prep, www.nu-chekprep.com) were used to optimize MS parameters. TAG species containing either EPA or DHA were first identified using ESI-MS/MS neutral loss survey scans corresponding to 319 m/z and 345 m/z (RCOOH + NH_3_), respectively (Figure S5A,B). Lipids were extracted from mature seeds as described above, and re-suspended in a solution of iso-propyl alcohol/methanol/50 mm ammonium acetate/dichloromethane (4:3:2:1 v/v) for direct infusion of the unfractionated lipids into the mass spectrometer. The fatty acid composition of individual TAG species identified in the survey scans was then established using enhanced product ion scans (Figure S5C,D). The amount of each TAG was expressed as a percentage of the total values for all TAG species. There is variation in ionization efficiency among acyl glycerol species with different fatty acyl groups, and no response factors for individual species were determined in this study, therefore the values are not directly proportional to the TAG content of each species. However, the approach does allow a realistic comparison of TAG species across samples. After injection of each single-seed TAG sample for LC-MS/MS analysis, the remaining 95 μl was then methylated and the methyl ester derivatives were analysed by GC-FID as described above.

### Acyl CoA analyses

Freshly harvested seeds were frozen in liquid nitrogen, and acyl CoAs were extracted as described by Larson and Graham (2001) and analysed using ESI-MS/MS + MRM or LC-MS/MS + MRM in positive ion mode. The LC-MS/MS + MRM analysis (using an ABSciex 4000 QTRAP) was performed as described by Haynes *et al*. (2008) (Agilent 1200 LC system; Gemini C18 column (Phenomenex), 2 mm inner diameter, 150 mm length, particle size 5 μm). For the purpose of identification and calibration, standard acyl CoA esters with acyl chain lengths from C14 to C20 were purchased from Sigma as free acids or lithium salts.

## Supporting Information

Figure S1 Comparison of *C. sativa* wild-type, RRes_EPA and RRes_DHA plants at 6 and 10 weeks old. **Figure S2.** Analysis of major phospholipid species in wild-type and engineered mature *C.sativa* seed. **Figure S3.** LC-MS/MS + MRM analysis of TAG from *C. sativa* engineered for production of EPA and/or DHA. **Figure S4.** Parallel analysis (GC-FID) of FAMEs derived from the single-seed LC-MS/MS TAG analysis. **Figure S5.** Application of mass spectrometry to identify TAG molecular species containing EPA and/or DHA.

Table S1 Total fatty acid composition of oil seeds from wild-type and transgenic *C. sativa* plants.
