# Supplementary material for: Successful high-level accumulation of fish oil omega-3 long-chain polyunsaturated fatty acids in a transgenic oilseed crop
Source: Plant J. 2013 Nov 8;77(2):198–208. doi: 10.1111/tpj.12378 (PMC4253037; doi:10.1111/tpj.12378)
Supplement: Figure S1 — Comparison of C. sativa wild-type, RRes_EPA and RRes_DHA plants at 6 and 10 weeks old. Figure S2. Analysis of major phospholipid species in wild-type and engineered mature C.sativa seed. Figure S3. LC-MS/MS + MRM analysis of TAG from C. sativa engineered for production of EPA and/or DHA. Figure S4. Parallel analysis (GC-FID) of FAMEs derived from the single-seed LC-MS/MS TAG analysis. Figure S5. Application of mass spectrometry to identify TAG molecular species containing EPA and/or DHA. [file tpj0077-0198-SD1.pdf]

Fig. S1

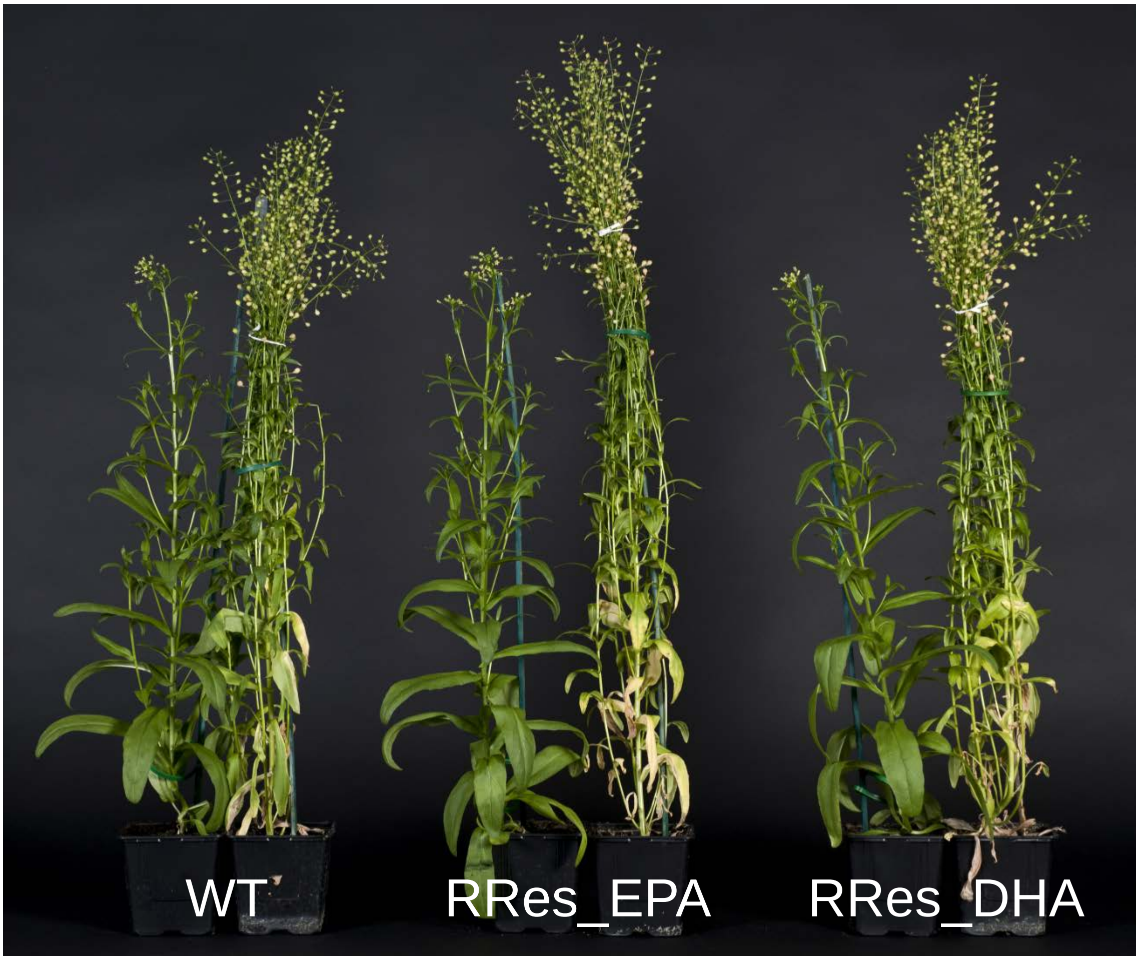

Figure S1. A comparison of *C. sativa* wild type, RRes_EPA and RRes_DHA, six and ten weeks old respectively.

No phenotypic differences are obvious

Fig S2.

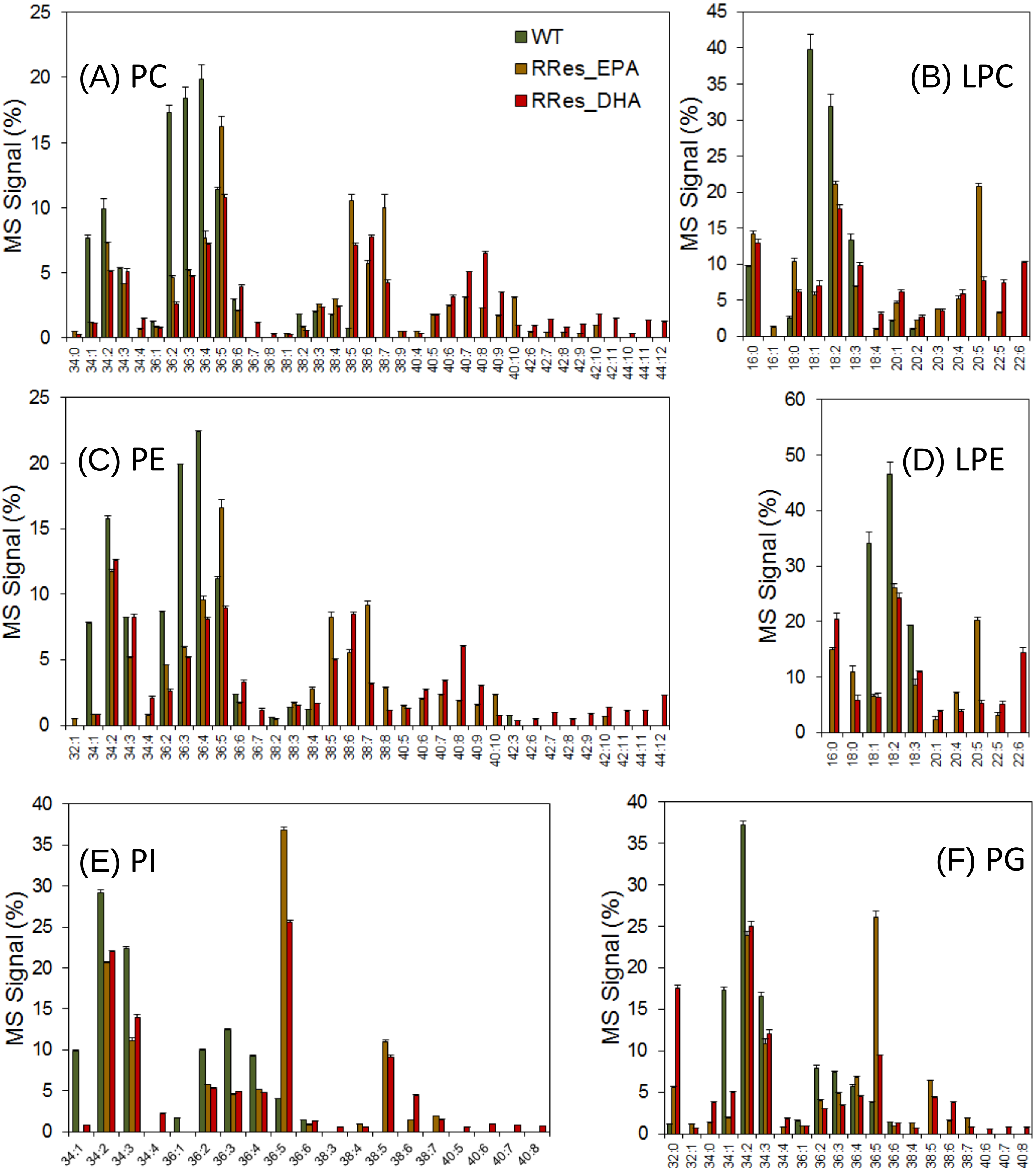

Figure S2. Analysis of major phospholipid species in wild type and engineered mature *C. sativa* seed. Values are means +/- SE (n= 3). (A) PC, phosphatidylcholine; (B) lyso-PC; (C) PE, phosphatidylethanolamine; (D) lyso-PE; (E) PI, phosphatidylinositol; (F) PG, phosphatidylglycerol.

Fig. S3

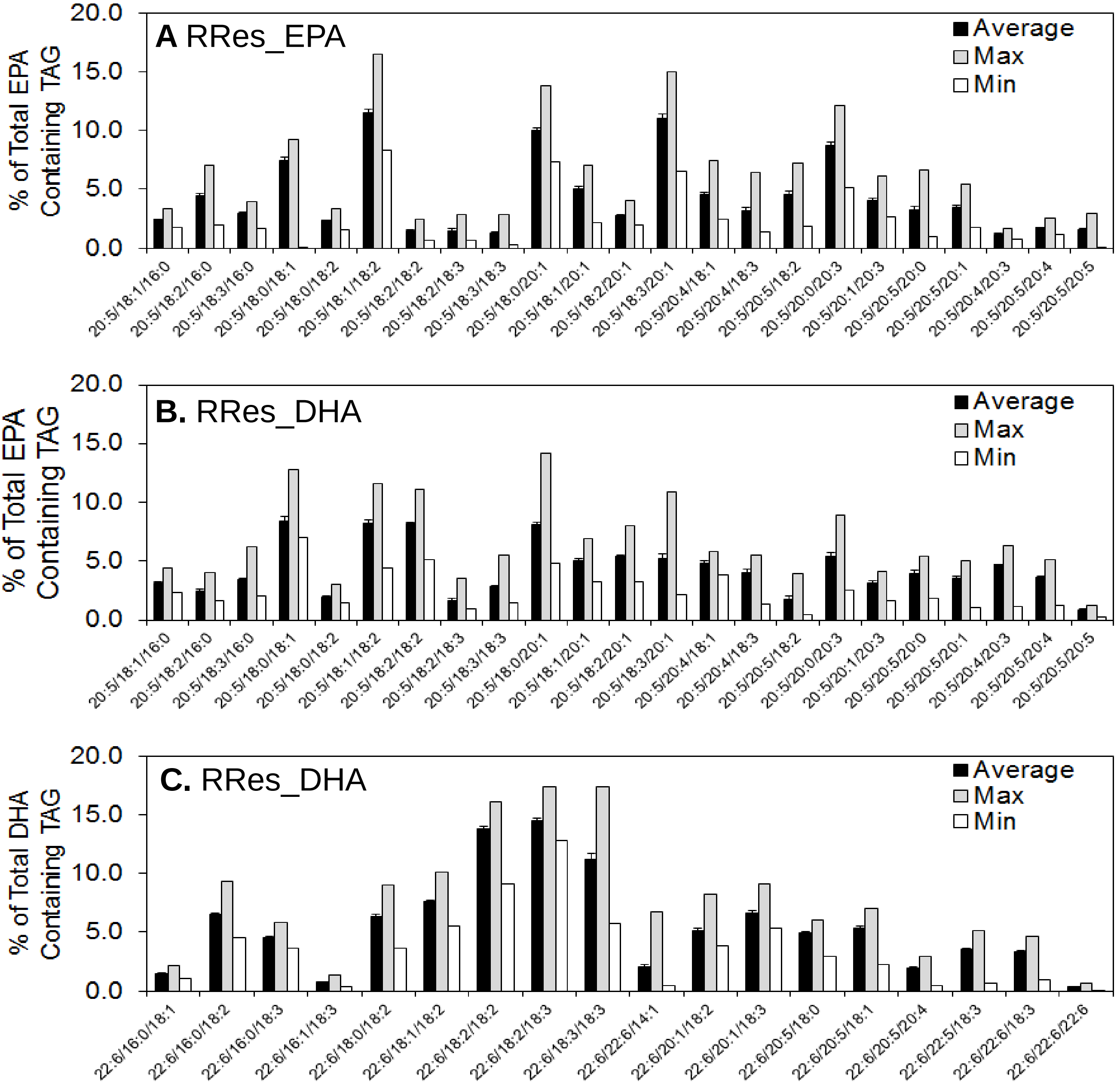

Figure S3. LC-MS/MS +MRM analysis of TAG from *C. sativa* engineered for the production of EPA and/or DHA. The average, maximum and minimum accumulation of the major TAG species in single seeds: (**A**) EPA-containing TAG in RRes_EPA; (**B**) EPA-containing TAG in RRes_DHA and (**C**) DHA-containing TAG in RRes_DHA.

Fig. S4

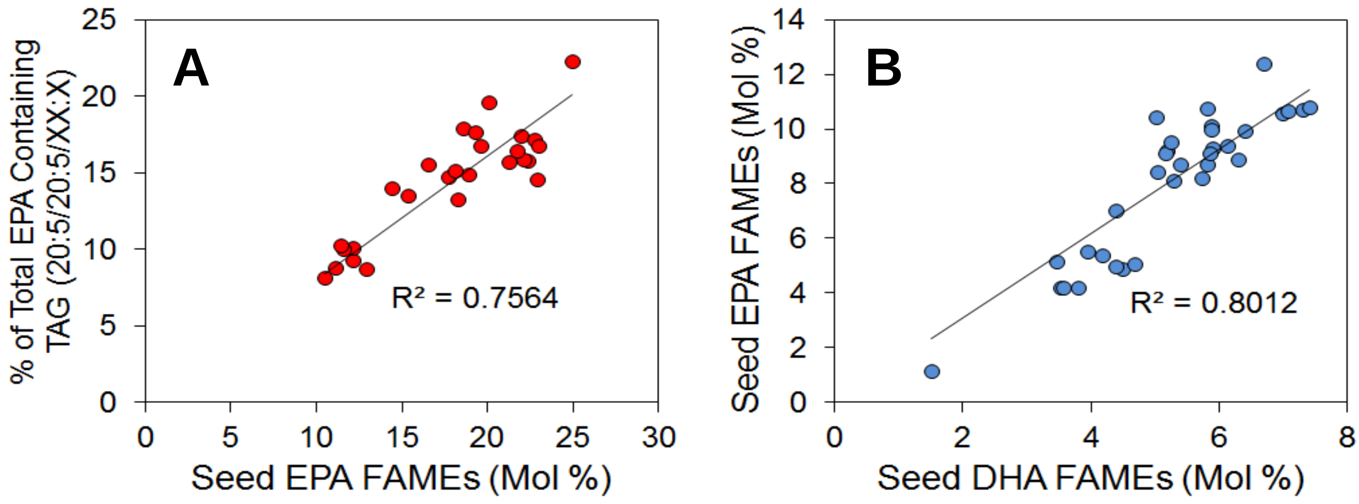

Figure S4. The parallel analysis (GC-FID) of FAMEs derived from the single seed LC-MS/MS TAG analysis. (**A**) The relationship in RRes_EPA seed between EPA content and TAG species containing two or more molecules of EPA. (**B**) The capacity of *C. sativa* RRes_DHA TAG to accumulate high levels of both EPA and DHA.

Fig. S5

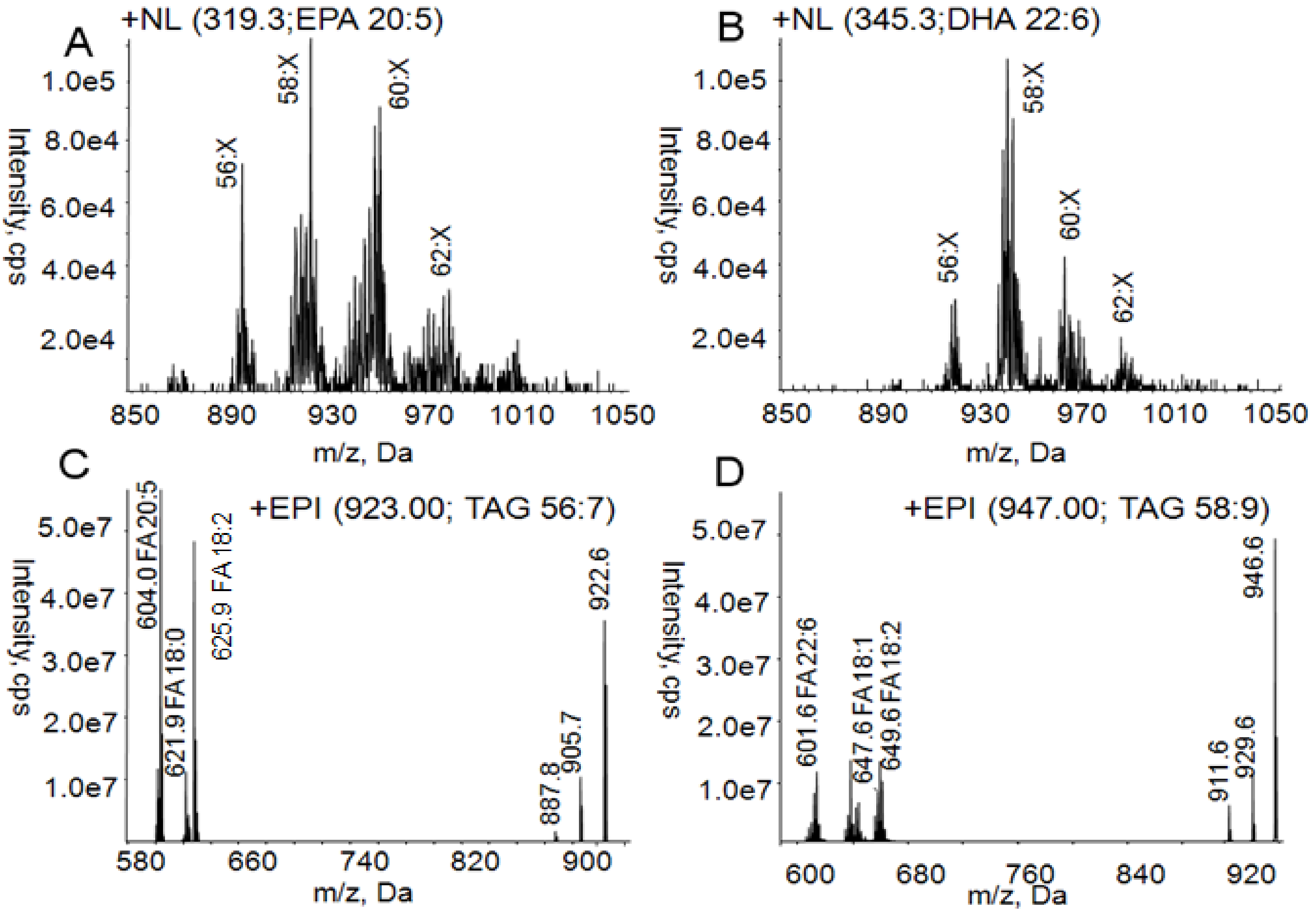

Figure S5. The application of mass spectrometry to identify TAG molecular species containing EPA and/or DHA. A direct-infusion electrospray ionization-tandem mass spectrometry approach was used characterise those TAG molecular species in seed oil containing EPA and/or DHA. Initial scans targeted the neutral loss of (**A**) EPA 319.3 m/z and (**B**) DHA 345.3 m/z. For each of the major TAG species identified, enhanced product ion scans were then used to determine the fatty acid composition, illustrated here for TAG 56:7 (**C**) and Tag 58:9 (**D**). This information was then used to design individual MRM for each TAG as described in the Experimental Procedures.
