# Supplementary material for: Successful high-level accumulation of fish oil omega-3 long-chain polyunsaturated fatty acids in a transgenic oilseed crop
Source: Plant J. 2013 Nov 8;77(2):198–208. doi: 10.1111/tpj.12378 (PMC4253037; doi:10.1111/tpj.12378)
Supplement: Table S1 — Total fatty acid composition of oil seeds from wild-type and transgenic C. sativa plants. [file tpj0077-0198-SD2.docx]

**Table S1** Total fatty acid composition (Mol%) of oilseeds from wild-type and transgenic plants.

|  | **Wt** |  | **EPA Lines** | |  | | **DHA Lines** | | |
| --- | --- | --- | --- | --- | --- | --- | --- | --- | --- |
|  |  |  | T2  (N=8) | T3  (N=199) | |  | | T2  (N=12) | T3  (N=15) |
| **16:0** | 6.5 ±0.1 |  | 8.5 ± 0.8 | 8.3 ± 0.6 | |  | | 8.9 ± 0.9 | 9.0 ± 0.4 |
| **18:0** | 3.4 ±0.1 |  | 4.7 ± 0.5 | 6.7 ± 0.9 | |  | | 4.9 ±0.8 | 4.6 ± 0.3 |
| **18:1** | 14.5 ±0.4 |  | 5.9 ± 1.6 | 5.0 ± 0.8 | |  | | 6.1 ± 1.8 | 7.4 ± 1.1 |
| **18:2** | 19.0 ±0.6 |  | 20.3 ± 2.3 | 20.6 ± 1.2 | |  | | 20.0 ± 2.1 | 20.4 ± 1.0 |
| **GLA** | - |  | 2.7 ± 1.8 | 2.4 ± 1.7 | |  | | 4.0 ± 1.7 | 5.2 ± 0.9 |
| **ALA** | 30.8 ±0.6 |  | 17.8 ± 5.4  (12.1 - 26.1) | 15.8 ± 2.9  (9.5 - 24.2) | |  | | 19.4 ± 3.2  (14.2 - 24.3) | 14.3 ± 1.1  (11.7 - 16.6) |
|  |  |  |  |  | |  | |  |  |
| **SDA** | - |  | 2.9 ± 1.2 | 1.5 ± 1.2 | |  | | 3.3 ± 1.3 | 4.3 ± 0.9 |
| **20:1** | 14.5 ±0.1 |  | 8.0 ± 1.8 | 7.0 ± 0.9 | |  | | 6.9 ± 1.4 | 6.8 ± 0.5 |
| **DGLA** | - |  | - 1. ± 0.4 | 1.1 ± 0.4 | |  | | 0.6 ± 0.3 | 0.8 ± 0.2 |
| **ARA** | - |  | 1.7 ± 0.7 | 1.6 ± 0.3 | |  | | 1.7 ± 0.5 | 2.2 ± 0.3 |
| **ETA** | - |  | 2.8 ± 1.6 | 3.2 ± 1.1 | |  | | 2.3 ± 0.8 | 2.0 ± 0.4 |
| **EPA** | - |  | 13.8 ± 4.1  (6.8 - 18.7) | 14.7 ± 2.8  (8.7 - 23.9) | |  | | 6.2 ± 2.1  (3.9 - 11.3) | 7.3 ± 1.4  (5.3 - 10.7) |
|  |  |  |  |  | |  | |  |  |
| **DPA** | - |  | - | - | |  | | 1.1 ± 0.5 | 1.6 ± 0.3 |
| **DHA** | - |  | - | - | |  | | 5.2 ± 1.5  (3.1 - 7.8) | 4.6 ± 0.7  (3.5 - 6.2) |
|  |  |  |  |  | |  | |  |  |
| **Others** | 11.2 ±0.1 |  | 10.1 ± 2.3 | 11.9 ± 1.0 | |  | | 9.3 ± 0.6 | 9.5 ± 0.4 |
|  |  |  |  |  | |  | |  |  |

Note for T2 N = refers to number of transgenic events analysed, whilst for T3 it is a mixture of events and plants. Each value represents the mean ± SD. The values in brackets indicate the fatty acid ranges for ALA, EPA and DHA. Other fatty acids include 16:1, 20:0, 20:3, 22:0, 22:1, 24:0, 24:1 and others
