# Supplementary material for: Successful high-level accumulation of fish oil omega-3 long-chain polyunsaturated fatty acids in a transgenic oilseed crop
Source: Plant J. 2013 Nov 8;77(2):198–208. doi: 10.1111/tpj.12378 (PMC4253037; doi:10.1111/tpj.12378)
Supplement: Supplementary file 3 [file tpj0077-0198-SD3.docx]

**SUPPLEMENTAL INFORMATION**

**Figure S1.** **A comparison of *C. sativa* wild type, RRes_EPA and RRes_DHA, six and ten weeks old respectively.** No phenotypic differences are apparent.

**Figure S2.** **Analysis of major phospholipid species in wild type and engineered mature *C.sativa* seed.** Values are means +/- SE (n= 3). (a) PC, phosphatidylcholine; (b) lyso-PC; (c) PE, phosphatidylethanolamine; (d) lyso-PE; (e) PI, phosphatidylinositol; (f) PG, phosphatidylglycerol.

**Figure S3. LC-MS/MS +MRM analysis of TAG from *C. sativa* engineered for the production of EPA and/or DHA.** The average, maximum and minimum accumulation of the major TAG species in single seeds: (**A**) EPA-containing TAG in RRes_EPA; (**B**) EPA-containing TAG in RRes_DHA and (**C**) DHA-containing TAG in RRes_DHA.

**Figure S4.** **The parallel analysis (GC-FID) of FAMEs derived from the single seed LC-MS/MS TAG analysis.** (**A**) The relationship in RRes_EPA seed between EPA content and TAG species containing two or more molecules of EPA. (**B**) The capacity of *C. sativa* RRes_DHA TAG to accumulate high levels of both EPA and DHA.

**Figure S5. The application of mass spectrometry to identify TAG molecular species containing EPA and/or DHA.** A direct-infusion electrospray ionization-tandem mass spectrometry approach was used characterise those TAG molecular species in seed oil containing EPA and/or DHA. Initial scans targeted the neutral loss of (**A**) EPA 319.3 m/z and (**B**) DHA 345.3 m/z. For each of the major TAG species identified, enhanced product ion scans were then used to determine the fatty acid composition, illustrated here for TAG 56:7 (**C**) and Tag 58:9 (**D**). This information was then used to design individual MRM for each TAG as described in the Experimental Procedures.

**Table S1**. Total fatty acid composition (Mol%) of oil seeds from wild type and transgenic *C. sativa* plants.
